# Invited Response on: Letter to the Editor: ‘‘Standardized Three-Dimensional Lateral Distraction Test: Its Reliability to Assess Medial Canthal Tendon Laxity’’

**DOI:** 10.1007/s00266-021-02586-9

**Published:** 2021-10-04

**Authors:** Xiaoyi Hou, Alexander C. Rokohl, Yongwei Guo, Ludwig M. Heindl

**Affiliations:** 1 Department of Ophthalmology, Faculty of Medicine and University Hospital Cologne, University of Cologne, Kerpener Strasse 62, 50937 Cologne, Germany; 2 Center for Integrated Oncology (CIO), Aachen-Bonn-Cologne-Duesseldorf, Cologne, Germany

*Level of Evidence V:* This journal requires that authors assign a level of evidence to each article. For a full description of these Evidence-Based Medicine ratings, please refer to the Table of Contents or the online Instructions to Authors www.springer.com/00266.

We very much welcome the correspondence by our highly esteemed colleagues Shuai Yue and Mengran Ju [1]. We appreciate the opportunity to discuss these critical points raised by their comments.

In the study *Standardized Three-Dimensional Lateral Distraction Test: Its Reliability to Assess Medial Canthal Tendon Laxity* [2, 3], two observers coordinated the acquisition of the 3D image, while the lateral distraction test (LDT) was performed to detect simultaneously the displacement of the inferior punctum (Pu) and avoid influencing factors, such as the contraction of adjacent muscles. Furthermore, new images were captured when the observers noted shifted lower punctum position due to contraction of adjacent muscles before or during the LDT.

At first glance, botulinum toxin type A might be an option to avoid measurement errors theoretically by relaxing the adjacent muscles. However, botulinum toxin type A also influences the measurements since the natural physiological state of the lower eyelid might change due to tone loss of these adjacent muscles. The punctum might be displaced and everted or inverted without support from these adjacent muscles, such as the orbicularis oculi muscle. Furthermore, the invasive application procedure, potential complications, and side effects, dosage selection, long-term metabolic cycle (3–6 months), and patient satisfaction are also issues that need to be considered in addition to ethical concerns.

We also noted that the upper eyelid might cover the medial corneoscleral limbus in the neutral as well as the distracted position, especially in elderly participants with a flabby upper eyelid. We fully agree that further research has to be conducted to address this issue in detail. Using a computer system [4] to simulate the entire iris and pupil morphology according to the exposed iris for optimized detection of the pupil center and the corresponding limbus might be a potential solution. The two vertical lines passing through could be obtained by the mimic pupil center and the limbus point.

We sincerely apologize for the legends and label in Fig. 2. The legends should be corrected as: The position is recorded with ‘positive (+)’ if the pu’ is lateralized to the vertical line through the medial corneoscleral limbus. The position is recorded with ‘negative (-)’ if the pu’ is medialized to the vertical line through the medial corneoscleral limbus. Furthermore, the (-) and (+) labels should be switched on the right eye.

In summary, in our study, we proposed a novel and reliable 3D-LDT method for an easy evaluation of the MCT laxity that might be useful for preoperative evaluation and postoperative follow-up examinations [3].

## Declaration

### Human or animal participants

This article does not contain any studies with human participants or animals performed by any of the authors.
